# Whole genome analysis of infectious bovine keratoconjunctivitis in Angus cattle using Bayesian threshold models

**DOI:** 10.1186/1753-6561-5-S4-S22

**Published:** 2011-06-03

**Authors:** Kadir Kizilkaya, Richard G Tait, Dorian J Garrick, Rohan L Fernando, James M Reecy

**Affiliations:** 1 Department of Animal Science, Iowa State University, Ames, IA 50011 USA; 2 Department of Animal Science, Adnan Menderes University, Aydin 09100 Turkey; 3 Institute of Veterinary, Animal and Biomedical Sciences, Massey University, Palmerston North, New Zealand

## Abstract

Infectious bovine keratoconjunctivitis (IBK), also known as pinkeye, is characterized by damage to the cornea and is an economically important, lowly heritable, categorical disease trait in beef cattle. Scores of eye damage were collected at weaning on 858 Angus cattle. SNP genotypes for each animal were obtained from BovineSNP50 Infinium-beadchips. Simultaneous associations of all SNP with IBK phenotype were determined using Bayes-C that treats SNP effects as random with equal variance for an assumed fraction (*π*=0.999) of SNP having no effect on IBK scores. Bayes-C threshold models were used to estimate SNP effects by classifying IBK into two, three or nine ordered categories. Magnitudes of genetic variances estimated in localized regions across the genome indicated that SNP within the most informative regions accounted for much of the genetic variance of IBK and pointed out some degree of association to IBK. There are many candidate genes in these regions which could include a gene or group of genes associated with bacterial disease in cattle.

## Background

Infectious Bovine Keratoconjunctivitis (IBK), commonly known as pinkeye, is a contagious bacterial disease caused in beef cattle by *Moraxella bovis*. IBK is characterized by excessive tearing, inflammation of the conjunctiva, and ulceration of the cornea. In severe cases, perforation of the cornea may occur which leads to permanent blindness. IBK is considered the most important ocular disease in cattle, due to the decreased performance of infected individuals and its subsequent economic effects. IBK is an economically important, lowly heritable (0.10 - 0.25), categorical disease trait. Breed differences in susceptibility of IBK have been established. In a number of studies, Hereford, Jersey and Holstein breeds appear to be more susceptible to infection than *Bos Indicus* breeds [1,2]. Additive genetic effects within breed are also known to influence resistance to pinkeye [2]. Putative QTL on Chromosomes 1 (66 to 110 cM) and 20 (2 to 35 cM) have been reported [3] to be associated with IBK.

The objective of the current study was to detect single-nucleotide polymorphism (SNP) markers in linkage disequilibrium (LD) with QTL associated with IBK in Angus cattle.

## Material and Methods

### Phenotypic data

**Figure 1:**
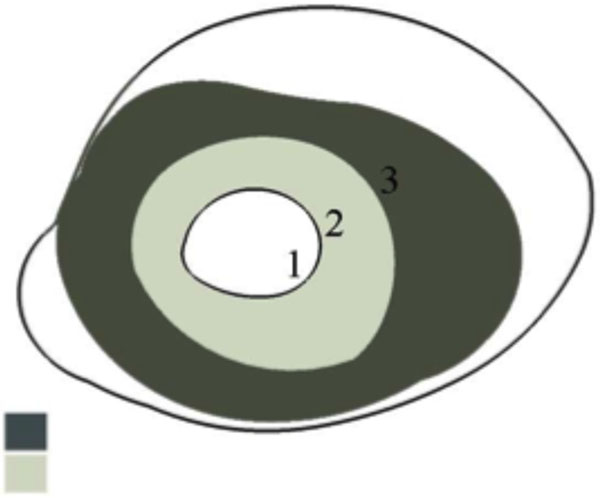
Illustration of the criteria used to assign severity scores to affected eyes. Score 1= ocular lesion covering less than 1/3 of the cornea; Score 2= ocular lesion covering 1/3 - 2/3 of the cornea; Score 3= ocular lesion covering more than 2/3 of the cornea

Infectious bovine keratoconjunctivitis records were collected from 858 animals born and raised in the Iowa State University Angus research herd. Both eyes were scored individually. The IBK scoring system for one eye used a scale from 0 to 4. A score of 0 denotes a cornea with no apparent lesions, 1 for a cornea with a lesion covering less than 1/3 of the cornea, 2 for a lesion covering 1/3 to 2/3 of the cornea, 3 for a lesion covering more than 2/3 of the cornea, and 4 for perforation of the cornea (Figure 1) [4].

Eye scores were pooled in various manners for categorical analysis as follows:

**IBK classified in two categories:** Incidence was scored as 0 for both unaffected eyes (63.7 %) and as 1 otherwise (37.3 %).

**IBK classified in three categories:** Incidence was scored as 0 for both unaffected eyes (63.7 %), 1 for single affected eye (26.4 %) and 2 for both affected eyes (10.9 %).

**IBK classified in nine categories:** Incidence was scored from 0 (63.7 %) to 8 (0.58 %) by adding the scores of the left and right eyes to utilize more information. For example, an animal with right eye score of 2 and a left eye score of 1 was scored as a 3.

### 50k SNP data

High-density SNP genotypes were obtained using the Bovine SNP50 Infinium II BeadChip (Illumina, Inc., San Diago CA). The Illumina A/B allele calls were used to compute a covariate for each locus that had values 0, 1, or 2 representing the number of B alleles. Missing genotypes represented less than 0.2% of total observations and were replaced with average covariate values. All genotypes were retained regardless of minor allele frequency.

### Threshold model

The Bayes-C model described by Kizilkaya et al., [5] was modified for categorical data analysis, and assumed a maker based bi- or multinomial threshold model for the underlying distribution of IBK (*l_c_i__*):(1)

where *l_c_i__* is the underlying liability to IBK (classified into two, three or nine categories, *c* = 2, 3 or 9) of individual *i*; **b** is a vector of contemporary group effects defined as the interaction of year and pasture management group; **x***_i_* is an incidence vector for **b**; *K* is the number of SNP marker loci; *Z_ij_* is the covariate at locus *j* for individual *i*; *β_j_* is the random substitution effect for locus *j*, which is conditional on  and assumed normally distributed  when *δ_j_*=1 but *β_j_*=0 when *δ_j_*=0, *δ_j_* is a random 0/1 variable indicating the absence (*δ_j_*=*0*) with probability *π* or presence (*δ_j_*=1) with probability 1 — *π* of locus *j* in the model; and e*_i_* is the random residual effect assumed normally distributed with *µ*=0 and known .

The Bayes-C threshold model was implemented in *GenSel* software [6] by assuming a fraction (*π*=0.999) of SNP markers have no effect on IBK scores. In the Markov Chain Monte Carlo (MCMC) implementation, a burn-in period of 5,000 MCMC cycles was used before saving samples from each of an additional 40,000 MCMC cycles. SNP marker effects *β_j_* were estimated by computing Monte-Carlo means of the posterior distribution of these effects using a Gibbs sampling strategy described in Kizilkaya et al., [5].

## Results and Discussion

**Figure 2:**
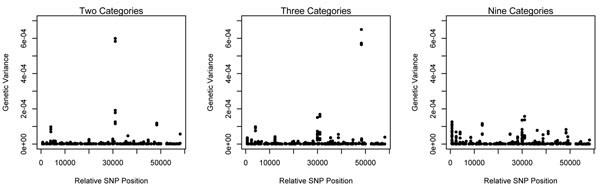
Genetic variances determined in the chromosomal regions ordered by map position from chromosome 1 to X defined by sliding windows of five consecutive SNP through the whole genome for IBK score classified into two, three or nine categories.

Genetic variances explained by SNP were calculated in the chromosomal regions defined by 5 contiguous SNP sliding windows. The 1000 largest genetic variance windows across the genome are shown in Figure 2 by plotting them with respect to their genomic locations (relative SNP positions). For IBK scored in two, three or nine categories, each categorisation indicated different degree of association to IBK in certain genomic regions. Furthermore, it appears that certain chromosomal regions on the genome had different associations according to the categorisation scale.

The regions on chromosomes 2 (4000, relative SNP position), 13 (30900) and 23 (48300) were found to be common across threshold model analyses and to have the highest genetic variances in the whole genome analysis of IBK scores for two, three or nine categories. Thereby, these regions could be indicative of QTL locations which are associated with IBK.

Comparison of genetic variances from analyses of IBK indicated that analysis of IBK scored into nine categories attributed smaller genetic variances to the regions on chromosomes 2, 13 and 23 than those with IBK defined in two and three categories. However, results from the nine category analysis additionally identified regions on chromosomes 1 and 20 which accounted for more genetic variance than these regions from analyses of IBK with two and three categories. SNP within the regions on chromosomes 1 and 20 were also found to reside within QTL regions identified by Casas and Stone [3] and within the QTL region on chromosome 20 related to incidence of pathogenic disease that include IBK, bovine respiratory disease, and infectious pododermatitis [7]. Furthermore, SNP within the regions identified in the present study were in regions of QTL for somatic cell count [8].

## Conclusions

Whole genome analysis of IBK using a marker-based threshold model identified genomic regions that were associated with IBK. Degree of associations determined in the analyses of IBK with two, three or nine classification were different for regions on chromosome 2, 13 and 23, which resulted from varying power across threshold model analyses depending on different number of observations in IBK categories with two, three or nine classification. Analysis of IBK with nine categories estimated smaller genetic variances for certain regions; however, it seemed to compensate for the loss of information by providing more indicative regions of QTL across the genome, especially on chromosomes 1 and 20. Analyses herein also provided confirmatory evidence of SNP associated with putative QTL on chromosome 1 and 20 for IBK. These findings should motivate future studies in whole genome analysis to identify the genetic basis of IBK.

## Competing interests

The authors declare that they have no competing interests.

## Authors’ contributions

KK developed the model and part of the program, and carried out the statistical analysis. RGT did the data preparation and helped with the statistical analysis. DJG and RLF developed the GenSel program used for analysis and JMR conceived the study, participated in its design and coordination. KK, RGT, DJG, RLF and JMR helped to draft the manuscript. All authors read and approved the final manuscript.
